# Detection of circulating tumor cells in the cerebrospinal fluid of a patient with a solitary metastasis from breast cancer: A case report

**DOI:** 10.3892/ol.2014.1993

**Published:** 2014-03-24

**Authors:** AKSHAL S. PATEL, JOSHUA E. ALLEN, DAVID T. DICKER, JONAS M. SHEEHAN, MICHAEL J. GLANTZ, WAFIK S. EL-DEIRY

**Affiliations:** 1 Laboratory of Translational Oncology and Experimental Cancer Therapeutics, Department of Medicine (Hematology/Oncology), Penn State Hershey Cancer Institute, Penn State College of Medicine, Hershey, PA 17033, USA; 2 Department of Neurological Surgery, Penn State Hershey Medical Center, Hershey, PA 17033, USA

**Keywords:** cerebrospinal fluid, brain metastasis, leptomeningeal disease, metastatic breast cancer, brain lesion

## Abstract

Brain lesions identified following the diagnosis and eradication of primary cancers are often ambiguous in origin, existing as a solitary metastasis or an independent primary brain tumor. The brain is a relatively common site of metastasis with breast cancer, although determining whether metastases have originated from the breast or brain is often not possible without invasive biopsies. In the current case report, a patient presented with a brain lesion identified by radiography and was without systemic disease. The patient had previously exhibited a complete response to chemotherapy and surgery for a poorly differentiated invasive ductal carcinoma. The origin of the brain lesion could not be determined by magnetic resonance imaging, giving rise to a diagnostic dilemma with diverging treatment options. We previously reported a method to isolate and enumerate tumor cells of epithelial origin in the cerebrospinal fluid (CSF). CSF tumor cell analysis of the patient revealed massive CSF tumor cell burden of epithelial origin, indicating that the brain lesion was likely of breast origin. The current case report highlights the use of CSF tumor cell detection as a differential diagnostic tool, in addition to its previously demonstrated use as a marker of disease burden and therapeutic response.

## Introduction

There are two divergent treatment pathways for the comprehensive treatment of brain metastases and primary glioneuronal tumors. The two tumor types must be considered in the context of the overall clinical status of the patient, for example the patient’s age and neurological function. In the case of metastatic tumors, the systemic disease burden, number of cerebral lesions and their location must also be taken into account. With glial tumors, radiological findings may provide specific information indicating the tumor grade, but definitive diagnosis and classification by the World Health Organization criteria requires, at a minimum, tissue biopsy for histopathological examination (1). Only after pathological confirmation may an appropriate plan of treatment be formulated based on classification criteria (2).

Certain primary brain tumors have shown responses to chemotherapeutic agents, for example temozolomide, that are highly dependent on the methylation status of tumor-associated genes, including O^6^-methylguanine-DNA methyltransferase (3,4). The role of cytoreductive surgery in the management of a number of gliomas remains the subject of debate, however, surgery is often recommended for management of tumor-related mass effects (5,6). Fractionated radiotherapy remains the mainstay of radiation-based treatment, due to the infiltrative nature of glioneuronal tumors. By contrast, metastatic tumors are often treated using a multimodal approach that includes surgery, stereotactic radiosurgery and radiotherapy, with a much smaller role for chemotherapy (7,8). The role of radiosurgery in the management of metastatic tumors has increased exponentially in the past 15 years, in response to experience and success with this mode of treatment. Microsurgery is usually reserved for surgically accessible symptomatic lesions causing clinically significant mass effects. The use of microsurgery alone leads to unacceptably high local failure rates and radiotherapy is often necessary in conjunction with surgery for treating metastatic tumors. Considering the markedly different treatment options for these diseases, a clear differential diagnosis is necessary to provide the appropriate clinical care. This study was deemed to be non-human research by the Institutional Review Board of Penn State Hershey Medical Center (Hershey, USA). Standard procedural consent was obtained from the patient for the blood draw and lumbar puncture.

## Case report

A 36-year-old Caucasian female identified a lump in the upper outer quadrant of the left breast and axilla in February 2011. An ultrasound-guided left breast and lymph node biopsy in March revealed the presence of an estrogen receptor-positive and progesterone- and human epidermal growth factor receptor 2-negative poorly differentiated invasive ductal carcinoma. Additional immunohistochemical study results were consistent with a basal-like phenotype and the patient was found to have an IVS12+2del21 mutation in the breast cancer 1 (BRCA1) gene. Dose-dense adriamycin and cytoxan, followed by dose-dense paclitaxel (the two regimens every 2 weeks), were administered between April and August 2011. Following completion of chemotherapy, the patient underwent mastectomy of the left breast, axillary lymph node dissection and prophylactic right mastectomy in September 2011. A complete pathological response was observed.

**Figure 1:**
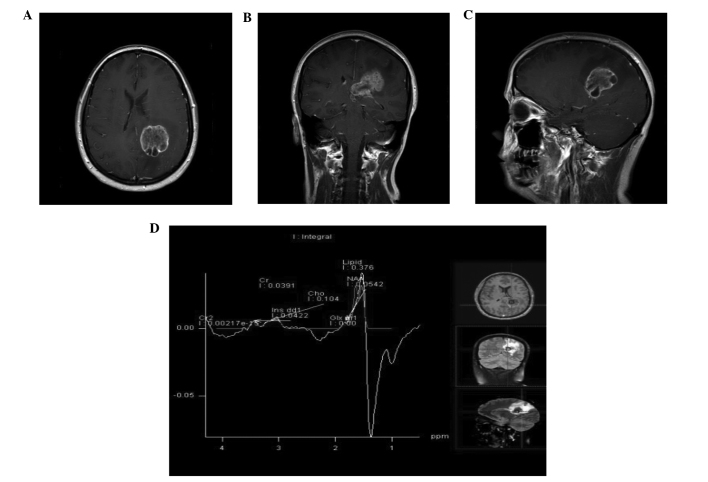
Radiographic imaging and infrared spectroscopy of a solitary brain lesion in a breast cancer patient. (A) Transverse, (B) coronal and (C) sagittal gadolinium-enhanced magnetic resonance images revealing a solitary brain lesion. (D) Near-infrared spectroscopy analysis of the brain lesions.

Postoperatively, chest wall radiation and a bilateral oophorectomy were performed. In early April 2012, the patient began to experience increasingly severe occipital and neck pain, followed by increasingly prominent impairment of balance and gait. Gadolinium-enhanced magnetic resonance imaging of the brain revealed a large (4.4×3.2×4.2 cm) peripherally enhancing and centrally necrotic mass centered in the left parietal lobe, extending across the corpus callosum into the right hemisphere (Fig. 1). A positive emission tomography-computed tomography scan demonstrated no evidence of disease outside the nervous system. Based on the lesion’s radiographic appearance, the neuroradiologist hypothesized that it may represent a solitary metastasis or a malignant primary glioma.

The brain is a relatively common site of metastasis for breast cancer and patients with a BRCA1-mutant form may be more likely to develop brain metastases than non-carriers (9). By applying a well-validated nomogram, designed to predict the likelihood of subsequent brain metastases in patients with metastatic breast cancer (10), the current case report estimated the risk of brain metastases in the patient to be ~14%. The patient was referred to radiation oncology and neurosurgery. Given the vastly different treatment algorithms for metastatic breast cancer and malignant gliomas, confirmation of the nature of the mass was requested. Magnetic resonance spectroscopy was performed, but was unable to definitively resolve this ambiguity.

In an attempt to shed light on this diagnostic dilemma, the patient’s blood and cerebrospinal fluid (CSF) were tested for the presence of circulating tumor cells (CTCs) with the CellSearch system (Janssen Diagnostics LLC, South Raritan, NJ, USA), using standard and adapted instructions reported previously (11). This technique requires expression of the epithelial cell adhesion molecule (EpCAM) for detection, which is not expressed by glial cells. Therefore, tumor cells of glial origin are not detected by this method. However, breast tumor cells are readily detectable, as previously demonstrated (11). CTCs were not detected in the peripheral blood of the patient but 6,703 EpCAM-positive tumor cells in 9 ml CSF were detected. This strongly suggests that the tumor was of epithelial rather than glial origin.

The patient underwent two lumbar punctures and pathology confirmed the presence of malignant cells by cytological examination. Next, the patient received fractionated external beam radiation to the lesion, placement of a right frontal ventricular access device and intrathecal chemotherapy. The initial agents used were liposomal cytarabine, topotecan, methotrexate and etoposide. Under conditions where a clear diagnosis was not made, the patient may have undergone two very different treatment algorithms, particularly with regards to chemotherapy and radiation.

## Discussion

The biology of CTCs remains an active area of study. The early stages of cancer spread may be due to micro-metastases seeding the blood and circulating until the appropriate ‘soil’ is found (12). Previous clinical studies have demonstrated the prognostic value of CTCs in several metastatic solid tumors*,* including breast cancer, as well as their use in monitoring tumor response to therapy (13,14). The CellSearch system is a fully automated system that relies on immunomagnetic enrichment with EpCAM-labeled iron oxide nanoparticles. Subsequent identification criteria include nuclear DNA and expression of cytokeratin and cluster of differentiation 45. The final step involves analysis of the cells by trained personnel to ensure that only cells with the appropriate cytomorphological characteristics are counted.

The CellSearch system was designed to use blood as the biological matrix, although a modification of the system utilizing CSF was previously reported (11). This adapted protocol permits tumor cells to be accurately and sensitively counted in the CSF of patients with breast cancer metastases in the central nervous system, without detecting glial cells. Previous studies have indicated that the number of tumor cells correlates with the magnitude of disease (11). The current case study demonstrates the use of a ‘liquid biopsy’ protocol to enumerate cells in the patient’s CSF to provide diagnostic information, in addition to the magnitude of metastatic disease burden. Thus, this case report has extended the clinical utility of the liquid biopsy technique.

In conclusion, the ability of the CellSearch system to detect cells of epithelial origin was utilized in order to resolve a diagnostic dilemma involving a patient with a brain lesion without systemic disease but with prior poorly differentiated invasive ductal carcinoma that completely resolved following surgery and treatment. Distinguishing the origins of brain lesions is particularly complex when the patient has had a prior systemic disease that responded completely to therapy, which increases the probability of the lesion representing a metastatic, rather than a primary tumor. The impact of utilizing this technique to distinguish the tumor origin, as was the case for this situation, is particularly high when different diagnoses lead to considerably different treatment options.
